# Thioredoxin H (TrxH) contributes to adversity adaptation and pathogenicity of *Edwardsiella piscicida*

**DOI:** 10.1186/s13567-019-0645-z

**Published:** 2019-04-15

**Authors:** Bi-ying Wang, Hui-qin Huang, Shuang Li, Ping Tang, Hao-fu Dai, Jian-an Xian, Dong-mei Sun, Yong-hua Hu

**Affiliations:** 1 College of Life Science and Technology, Heilongjiang Bayi Agricultural University, Daqing, 163319 China; 2 Institute of Tropical Bioscience and Biotechnology, Chinese Academy of Tropical Agricultural Sciences, Haikou, 571101 China; 3 Laboratory for Marine Biology and Biotechnology, Qingdao National Laboratory for Marine Science and Technology, Qingdao, China; 4 Yunnan Agricultural University, Kunming, Yunnan 650200 China; 5 Hainan Key Laboratory for Research and Development of Natural Products from Li Folk Medicine, Haikou, 571101 China; 6 Hainan Provincial Key Laboratory for Functional Components Research and Utilization of Marine Bio-resources, Haikou, 571101 China

## Abstract

Thioredoxins (Trxs) play an important role in defending against oxidative stress and keeping disulfide bonding correct to maintain protein function. *Edwardsiella piscicida*, a severe fish pathogen, has been shown to encode several thioredoxins including TrxA, TrxC, and TrxH, but their biological roles remain unknown. In this study, we characterized TrxH of *E. piscicida* (named TrxH_Ep_) and examined its expression and function. TrxH_Ep_ is composed of 125 residues and possesses typical thioredoxin H motifs. Expression of *trxH*_*Ep*_ was upregulated under conditions of oxidative stress, iron starvation, low pH, and during infection of host cells. *trxH*_*Ep*_ expression was also regulated by ferric uptake regulator (Fur), an important global regulatory of *E. piscicida*. Compared to the wild type TX01, a markerless *trxH*_*Ep*_ in-frame mutant strain TX01∆*trxH* exhibited markedly compromised tolerance of the pathogen to hydrogen peroxide, acid stress, and iron deficiency. Deletion of *trxH*_*Ep*_ significantly retarded bacterial biofilm growth and decreased resistance against serum killing. Pathogenicity analysis shows that the inactivation of *trxH*_*Ep*_ significantly impaired the ability of *E. piscicida* to invade host cells, reproduce in macrophages, and infect host tissues. Introduction of a trans-expressed *trxH* gene restored the lost virulence of TX01∆*trxH*. There is likely to be a complex relationship of functional complementation or expression regulation between TrxH and another two thioredoxins, TrxA and TrxC, of *E. piscicida*. This is the first functional report of TrxH in fish pathogens, and the findings suggest that TrxH_Ep_ is essential for coping with adverse circumstances and contributes to host infection of *E. piscicida*.

## Introduction

*Edwardsiella piscicida* (formerly included in *E. tarda*) [1, 2], one family member of Enterobacteriaceae, is a Gram-negative, motile, rod-shaped bacterium. It is a serious fish pathogen and has a broad host range that includes many species of economically important fish such as Japanese eel, flounder, turbot, red sea bream, tilapia, and channel catfish [3]. Edwardsiellosis, which is caused by *E. piscicida*, is one of the most severe diseases in aquaculture with freshwater as well as marine water supply, and has led to great economic losses in the aquaculture industry worldwide [4–7]. In addition to fish, *E. piscicida* is also pathogenic to humans and can cause gastrointestinal disorders and other diseases [8]. Since *E. piscicida* is an important pathogen of aquatic animals, more and more studies about *E. piscicida* are reported. A large number of virulence factors/systems, such as type III (T3SS) and type VI (T6SS) secretion systems, a two-component regulatory system, hemolysin, LuxS/AI-2 quorum sensing system, molecular chaperons and RNA-binding protein Hfq, ferric uptake regulator, lysozyme inhibitors, and so on, are known to be involved in *E. piscicida* stress resistance or pathogenicity [9–13]. Accumulating evidence has made clear that *E. piscicida* is an intracellular pathogen with the capacity to evade host immune defense [11, 14]. Recently, a study on intracellular trafficking pathways indicates a clear preference of *E. piscicida* for certain endocytic pathways and an involvement of endosomes, lysosomes, and cytoskeletons in the infection process [15]. Cheng et al. reported that this bacteria uses the tricarboxylic acid cycle to evade complement-mediated killing [16]. Another study showed that Sip2 in *E. piscicida* contributes to serum survival, acid resistance, intracellular replication, and host infection [17]. However, studies on the tolerance of *E. piscicida* to oxidative stress are limited.

Oxidative stress is a problem often encountered by most microorganisms. Reactive oxygen species (ROS) are abundantly generated in various environments including in host environments, leading microorganisms to develop multiple strategies to cope with oxidative stress [18]. One of these strategies is the thioredoxin system. The thioredoxin system is widely distributed in several species, from archaea and bacteria to humans, and is composed of thioredoxin (Trx), thioredoxin reductase (TrxR), and nicotinamide adenosine dinucleotide phosphate (NADP/NADPH) [19]. This antioxidant system transfers electrons from NADPH through TrxR to Trx and then to a large range of proteins that play critical roles in DNA synthesis and oxidative stress [20]. The major function of the Trx system is to reduce disulfide bridges in target proteins and help organisms recover from ROS stress [21, 22]. In microbes, such as *Escherichia coli*, *Bacillus subtilis*, *Staphylococcus aureus*, *Helicobacter pylori*, thioredoxins have been confirmed to involve in maintaining the intracellular redox homeostasis and play a key role in the protection of cells against ROS [23–25]. Moreover, the Trx system also provides the electron donor for many critical enzymes by its function of disulfide reductase and thus is related to a series of cellular functions [26].

Trx is a small (around 12 kDa) ubiquitous protein characterized by a conserved motif involving two redox-active Cys separated by a pair of amino acids (CxxC motif) [27]. Besides the oxidoreductase function, Trx has the function of regulating cell growth, inhibiting apoptosis and regulating gene transcription [28]. The *E. coli* genome encodes two Trx, Trx1 (TrxA), and Trx2 (TrxC). Trx1 is the first thioredoxin discovered in *E. coli*, and its function as a hydrogen donor in redox reactions was identified [29]. *H. pylori* also contains two genes that encode Trx, *trxA* and *trxC*. TrxA expression in *H. pylori* is induced by stress agents, and it is the electron donor for antioxidant enzymes [24, 30]. TrxC acts as a chaperone and transforms denatured arginase into its active form [31]. There are three thioredoxin in the *E. piscicida* genome, TrxA, TrxC, TrxH [32]. To our knowledge, there are no reports about the function study of TrxH in this pathogenic bacteria. In this study, we characterized TrxH in *E. piscicida* (named TrxH_Ep_), examined its expression profiles under different conditions, and analyzed its role in adversity and infection. Our results provide the first insights into the biological function of *E. piscicida* TrxH.

## Materials and methods

### Bacteria and growth conditions

*Escherichia coli* BL21 (DE3) was purchased from TransGen (Beijing, China). *E. coli* S17-1λpir was purchased from Biomedal (Sevilla, Spain). *E. piscicida* TX01 was isolated from diseased fish [33]. Bacteria were cultured in Luria–Bertani broth (LB) at 37 °C (for *E. coli*) or 28 °C (for *E. piscicida*). Where indicated, chloramphenicol, tetracycline, polymyxin B, and 2,2′dipyridyl (Dp) were supplemented at the concentrations of 30 μg/mL, 15 μg/mL, 100 μg/mL, and 100 μM respectively.

### Construction of the *trxH*_*Ep*_ mutation and its complementation

The primers used in this study are listed in Table 1. To construct the *trxH*_*Ep*_ knockout strain, TX01∆*trxH*, in-frame deletion of a 261 bp segment (residues 24 to 110) of *trxH*_*Ep*_ was performed by overlap extension PCR as follows: the first overlap PCR was performed with the primer pair TrxHF1/R1, the second overlap PCR was performed with the primer pair TrxHF2/R2, and the fusion PCR was performed with the primer pair TrxHF1/R2. The PCR products amplified by primer pair TrxHF1/R2 were inserted into the suicide plasmid pDM4 [34] at the Bgl II site, resulting in pDMTrxH. S17-1λpir was transformed with pDMTrxH, and the transformants were conjugated with TX01 as described previously [35]. The transconjugants were selected on LB agar plates supplemented with 10% sucrose. One of the colonies that were resistant to sucrose and sensitive to chloramphenicol (marker of pDM4) was analyzed by PCR, and the PCR products were subjected to DNA sequencing to confirm in-frame deletion. This strain was named TX01∆*trxH*. To construct the complementary strain TX01∆*trxH*C, *trxH*_*Ep*_ was amplified by PCR with primers TrxHF3/R3, and the following experimental operations were done as described previously [35].

**Table 1:** **Primers used in this study**

Primer	Sequences (5′ → 3′)
TrxhKOF1	GGATCCCTCAACTTGCCAATACCAT (BamHI)
TrxhKOR1	TCACCGATGCTAGCGGCCTGTACGG
TrxhKOF2	CCGCTAGCATCGGTGAAACGTCGCCA
TrxhKOR1	GGATCCATGCCGATCGCCATCAGC (BamHI)
TrxhF3	GGATCCCTGAGTATTGCCGAGC (BamHI)
TrxhR3	CATATGGCGGGTCAGAAAGTCAGAGA (HindIII)
TrxhRTF	AAACGGCAGGAGCGTGAA
TrxhRTR	GGGTGCTTTGCTGGCTGA
TrxARTF	GAGTATGCAGGGAAGCTGACCAT
TrxARTR	GGTTGGCATCCAGGAACTCTTT
TrxCRTF	TCCCGCCTGTCATACCACCA
TrxCRTR	GCGCCCAGAAGTCGATCAGTA
TrxhPF4	GATATCATTCAGCCCGGCAAAGAC (EcoRV)
TrxhPR4	GATATCATCCCACCTTTTTGTAATGTTC (EcoRV)

### Resistance to stressors (H_2_O_2_, iron deficiency and acidic pH) and to non-immune fish serum

TX01, TX01∆*trxH* and TX01∆*trxH*C were cultured in LB medium to exponential phase. To determine hydrogen peroxide and iron deficiency tolerance, LB agar plates (pH = 7) supplemented with or without 1 mM H_2_O_2_ or 50 μM 2,2′dipyridyl (Dp) were streaked with 5 μL TX01, TX01∆*trxH* and TX01∆*trxH*C, respectively. For acid resistance, LB agar plates with pH = 7 or pH = 5 were streaked with the three bacteria. The plate was incubated at 28 °C for 48 h, and bacterial growth was examined. For quantitative analysis, three strains were cultured in LB medium with three stress conditions for 24 h, then the populations of cultivated bacteria were counted by diluted plate. To determine hydrogen peroxide sensitivity, TX01 and TX01∆*trxH* were cultured in LB medium to exponential phase, then H_2_O_2_ was added to 5 mM, the bacterial growth was surveyed every hour. The experiment was performed three times.

TX01, TX01∆*trxH* and TX01∆*trxH*C were cultured in LB medium to exponential phase. Then the cells were washed with PBS and resuspended in PBS. Approximately 10^5^ bacterial cells were mixed with 50 μL fish serum or PBS (control). After incubation with mild agitation at 28 °C for 60 min, the mixtures were serially diluted and plated in triplicate on LB agar plates. The plates were incubated at 28 °C for 48 h, and the colonies that appeared on the plates were enumerated. The survival rate was calculated as follows: [(number of serum-treated cells)/(number of control cells)] × 100%. The experiment was performed three times.

### Biofilm assay

TX01, TX01∆*trxH* and TX01∆*trxH*C were cultured in LB medium to exponential phase and diluted to 10^5^ CFU/mL. The diluted cells were transferred into a 96-well polystyrene plate (Nunc, Denmark) and incubated at 28 °C for 24 h without agitation. Then the wells were washed gently five times with PBS. The attached cells were treated with Bouin fixative for 1 h and stained with 1% crystal violet solution for 20 min. After the treatment, the unbound dye was removed by rinsing the plate several times with running water. The plate was air dried. The bound dye was eluted in ethanol, and the *A*_570_ of eluates was measured. The experiment was performed three times.

### Invasion of eukaryotic cell lines

#### Interaction of bacteria with cultured fish cells

Examination of interactions between FG cells and *E. piscicida* was performed as described previously [36]. Briefly, TX01, TX01∆*trxH* and TX01∆*trxH*C were cultured in LB medium to exponential phase. FG cells were cultured in 96-well cell culture plates and 100 μL bacteria (1 × 10^7^ CFU/mL) were added to FG cells at a multiplicity of infection (MOI) of 10:1. After incubation at 23 °C for 0, 2, 4, 8 h, the plates were washed five times with PBS. To determine the number of bacterial cells associated with FG cells, the washed FG cells were lysed with 200 μL of 1% (vol/vol) Triton X-100 in PBS, and the lysate was diluted serially and was plated on LB agar plates supplemented with polymyxin B. After incubation overnight at 28 °C, the numbers of colonies that appeared on the plates were counted. For microscopy observation, TX01 and TX01∆*trxH* were labeled with FITC (Solarbio, Beijing, China). FG cells were cultured in Glass Bottom cell Culture Dish and infected with FITC-labeled bacteria for 4 h, the plates were washed three times with PBS. The cells were fixed by polyformaldehyde, then the fluorescence of extracellular bacteria was quenched by adding trypan blue (2 mg/mL in PBS). After washing three times with PBS, the cells were observed with a confocal microscope (Olympus Fluoview FV1000).

#### Bacterial replication in macrophages

The experiment was performed as described by Sui et al. [15] with slight modification. RAW264.7, a murine monocyte-macrophage cell line, were cultured in Dulbecco’s minimal Eagle’s medium (DMEM) (Gibco, USA) containing 10% fetal bovine serum (FBS) (Gibco, USA) at 37 °C in 5% CO_2_. Then 100 μL bacteria (1 × 10^7^ CFU/mL) were added to RAW264.7 cells at a multiplicity of infection (MOI) of 10:1, followed by incubation at 28 °C for 2 h. Extracellular *E. piscicida* was killed by adding gentamicin (100 μg/mL) to the plate, followed by incubation at 28 °C for 1 h. The cells were washed three times with PBS and cultured in DMEM containing 10 μg/mL gentamicin for 0, 2, 4, and 8 h. At each time point, the number of bacteria in macrophage was detected as described above. The experiment was performed three times.

### Fish and experimental challenges for bacterial dissemination in vivo

Clinically healthy Japanese flounder (*Paralichthys olivaceus*) (average 14.2 g) were purchased from a commercial fish farm in Shandong. The fish were maintained at ~22 °C in aerated seawater and fed daily with commercial dry pellets. Fish were acclimatized in the laboratory for 2 weeks before experimental manipulation. Before the experiment, fish were randomly sampled and examined for the presence of bacteria in the blood, liver, kidney, and spleen, and no bacteria were detected from the sampled fish. For tissue collection, fish were euthanized with an overdose of MS222 (tricaine methanesulfonate) (Sigma, USA) as previously described [37]. For tissue dissemination analysis, TX01, TX01∆*trxH*, and TX01∆*trxH*C were cultured in LB medium to an OD600 of 0.8. The cells were washed with PBS and resuspended in PBS to 10^6^ CFU/mL. Japanese flounder were divided randomly into four groups and infected by intramuscular (i.m.) injection with 50 μL TX01, TX01∆*trxH*, TX01∆*trxH*C, or PBS. The kidney and spleen were taken aseptically from the fish at 24 h and 48 h post-infection (hpi). Bacterial recovery from the tissues was determined as reported previously [35]. The rest of the infected fish were monitored daily for mortality for 20 days. The experiment was performed in triplicate.

### Quantitative real-time reverse transcriptase-PCR (RT-qPCR) analysis of *trxH*_*Ep*_, *trxA*, and *trxC* expression in wild TX01 strain and mutants under different environmental conditions

To examine *trxH*_*Ep*_ expression under in vitro conditions, TX01 was grown in LB medium with different pH (pH 5 or 7) at 28 °C, grown in LB medium with or without 2,2′dipyridyl at 28 °C, and grown in LB medium with or without hydrogen peroxide at 28 °C. The bacteria were harvested by centrifugation and total RNA was extracted with HP Total RNA kit (Omega Bio-Tek, USA). The RNA was treated with DNase with the kit of RNase-Free DNase Set (Omega Bio-Tek, USA). One microgram of total RNA was used for cDNA synthesis with the Superscript II reverse transcriptase (Invitrogen, Carlsbad, CA, USA). RT-qPCR were carried out as reported previously [35]. To examine *trxH*_*Ep*_ expression during infection of host cells, TX01 was cultured in LB medium to an OD_600_ of 0.8 and resuspended in PBS to 10^6^ CFU/mL. The resuspension was used to infect FG cells, a cell line derived from flounder gill cells, as described above [38]. The cells were harvested and used for total RNA extraction and RT-qPCR analysis of *trxH*_*Ep*_ expression as described above. The experiment was performed three times.

A *fur* mutant strain of *E. piscicida* was obtained in another study (data not published). The wild type *E. piscicida* TX01 and *fur* mutant strain were cultured in LB medium to the early exponential phase, then bacteria were harvested and total RNA was extracted. The expression of *trxH*_*Ep*_ expression in two strains were examined by RT-qPCR as described above.

To examine the expression of *trxA* and *trxC* in wild type *E. piscicida* and *trxH*_*Ep*_ knockout strain, TX01 and TX01∆*trxH* were cultured in LB medium with or without H_2_O_2_, cultured in LB medium with pH = 7 or pH = 5, and incubated with or without FG cells. The bacteria were harvested and total RNA was extracted. The expression of *trxA* and *trxC* expression in two strains were examined by RT-qPCR as described above.

### Transcriptional regulation of the *trxH*_*Ep*_ promoter by Fur

The speculative promoter of *trxH*_*Ep*_ (the 510 bp DNA upstream of *trxH*_*Ep*_ operon) was cloned by primers TrxhPF4/TrxhPR4 and was inserted into the SwaI site of pSC11, a promoter probe plasmid [39], resulting in pSH510. pSH510 was introduced into *E. coli* DH5α by transformation and cultured on X-gal (5-bromo-4-chloro-3-indolyl-beta-d-galactopyranoside) plate. DH5α/pSH510 was then transformed with pT (control) and pTFur, which expressed the Fur and was constructed as described by Wang et al. [40], and cultured on X-gal plate. The transformants were subjected to β-galactosidase assay [40].

### Statistical analysis

All statistical analyses were performed with SPSS 18.0 software (SPSS Inc., Chicago, IL, USA). Data were analyzed with analysis of variance (ANOVA), and statistical significance was defined as *P* < 0.05.

## Results

### Characterization of the *TrxH*_*Ep*_ sequence

**Figure 1:**
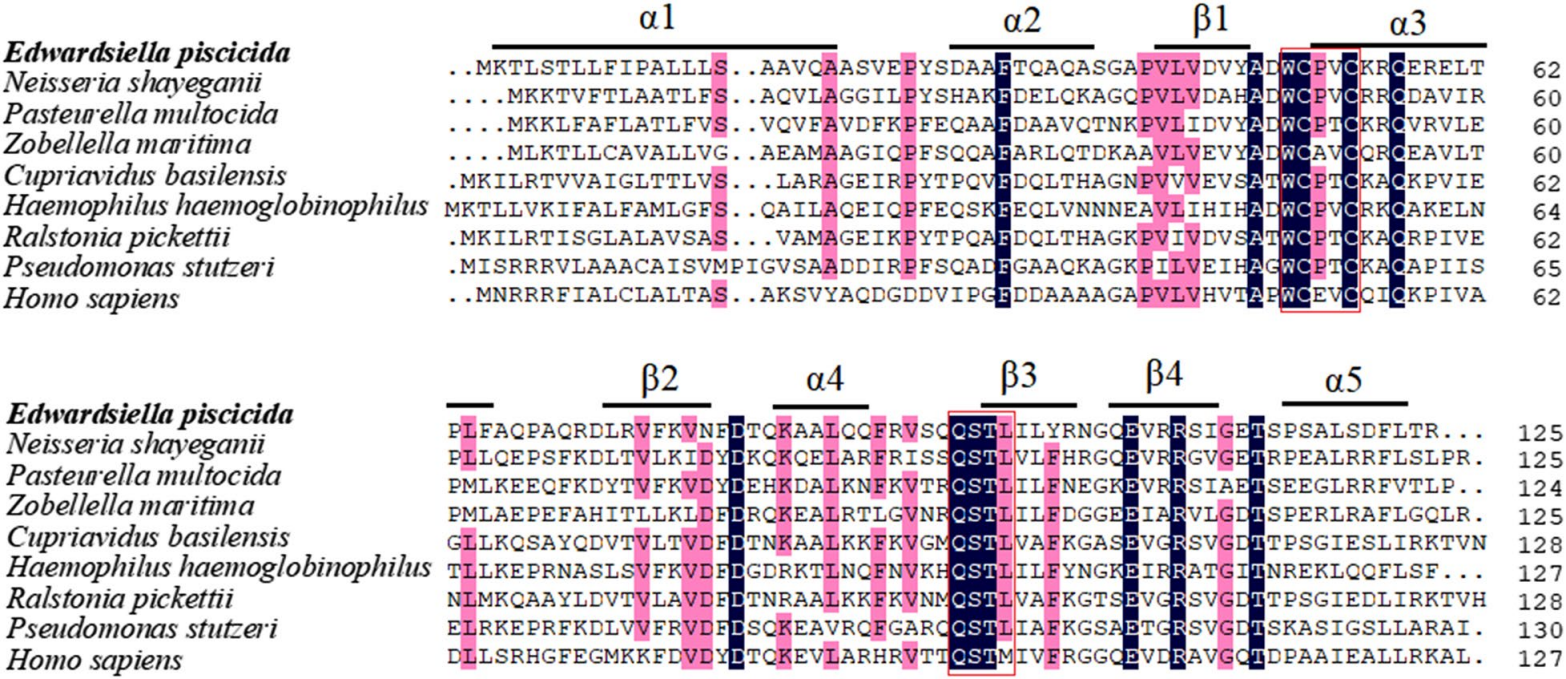
**Alignment of the amino acid sequences of TrxH**_**Ep**_ **homologues.** Dots denote gaps introduced for maximum matching. The consensus residues are in black, the residues that are ≥ 75% identical among the aligned sequences are in pink. Conserved WCXXC catalytic motif and QSTL/M motif are boxed. The characteristic elements of the thioredoxin fold (α1, α2, α3, α4, α5, β1, β2, β3, and β4) are underlined.

*trxH*_*Ep*_ codes for a putative protein of 125 amino acids, which contains a thioredoxin domain. According to BLAST sequence analyses, TrxH_Ep_ shares 49.6%–38.5% overall sequence identities with the thioredoxin homology of a number of species including *Neisseria shayeganii*, *Pasteurella multocida*, *Zobellella maritima*, *Cupriavidus basilensis*, *Haemophilus haemoglobinophilus*, *Ralstonia pickettii*, *Pseudomonas stutzeri*. Curiously, among the species with sequence identities of more than 30%, there are no common animal pathogenic bacteria even including *E. coli* (except *Edwardsiella*). TrxH_Ep_ also share sequence identity of 37.1% with thioredoxin of *Homo sapiens*. The WCXXC catalytic motif, the conserved active site of the TrxH family [41], exists in TrxH_Ep_, suggesting that TrxH_Ep_ is one member of the TrxH protein family. Another conservative motif, QSTL/M, also exists in TrxH_Ep_ (Figure 1). Protein secondary structure prediction shows that the characteristic elements of the thioredoxin fold (α1, α2, α3, α4, α5, β1, β2, β3, and β4) are found in TrxH_Ep_ (Figure 1).

### Mutation of *trxH*_*Ep*_ has multiple effects

To examine its functional importance, the *trxH* gene of *E. piscicida* TX01 was knocked out by markerless in-frame deletion of a region encoding amino acid residues 24 to 110. The resulting mutant was named TX01∆*trxH*. Meantime, the complementary strain TX01∆*trxH*C, which is a genetic variant of TX01∆*trxH* that expresses *trx* in trans from a plasmid, was constructed.

#### Effect on bacterial resistance against stressors

**Figure 2:**
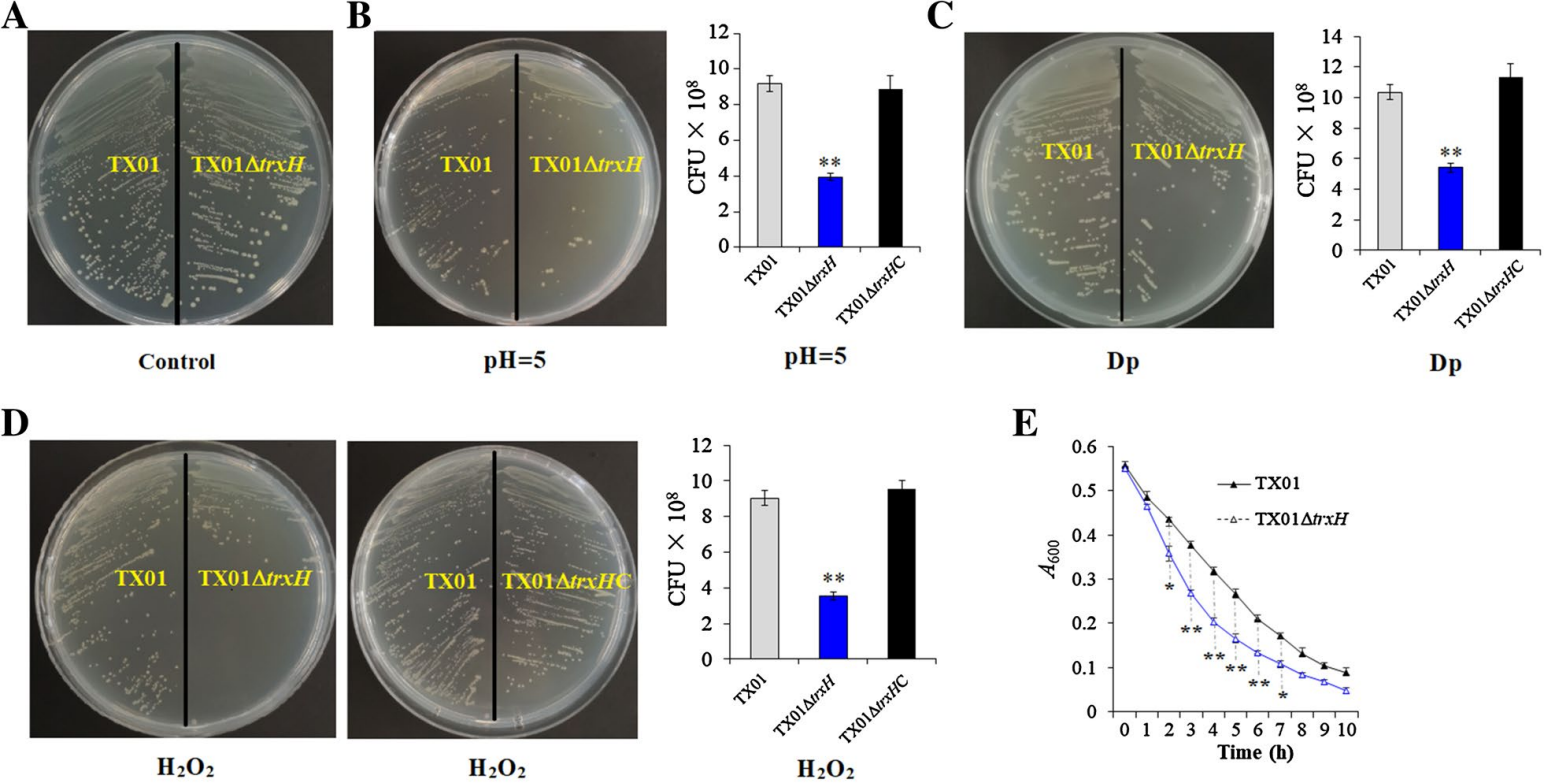
**Sensitivity of** ***Edwardsiella piscicida*** **to oxidative stress, iron deficiency, and acid stress.** TX01, TX01∆*trxH*, and TX01∆*trxH*C were cultured in LB medium and LB agar plates with pH = 7 (normal **A**), with pH = 5 (**B**), with 80 μM 2,2′dipyridyl (Dp, **C**), or with 1 mM H_2_O_2_ (**D**) at 28 °C for 24–48 h. The populations of cultivated bacteria were counted by diluted plate. **E** TX01 and TX01∆*trxH* were cultured to early logarithmic phase in normal LB medium, then 5 mM H_2_O_2_ were added to the medium, and cell density was measured at different time points by determining absorbance at OD_600_. Data are the means of three independent experiments and presented as mean ± SEM (*N* = 3). N, the number of times the experiment was performed. *P < 0.05, **P < 0.01.

Growth analysis shows that when cultured in LB medium or LB agar medium, TX01∆*trxH* and TX01 exhibited a comparative growth rate (Figure 2A and data not shown). This indicates that the *trxH*_*Ep*_ mutation had no effect on growth of *E. piscicida* in normal conditions. However, when cultured in an LB agar plate containing H_2_O_2_, TX01 grew normally, whereas TX01∆*trxH* hardly grew, and the amount of TX01∆*trxH* in H_2_O_2_ stress was significantly lower than TX01 (Figure 2D). Consistently, when bacteria grew to early logarithmic phase in LB medium and hydrogen peroxide was added to the medium, growth analysis shows that TX01∆*trxH* exhibited a higher sensitivity to H_2_O_2_ than TX01 at a subsequent growth period (Figure 2E). When cultured under acidic condition, TX01∆*trxH* grew poorer than TX01 and the amount of TX01∆*trxH* was significantly lower than that of TX01 (Figure 2B). Similarly, when cultured under iron deficiency, the growth of TX01∆*trxH* was obviously retarded, compared to TX01 (Figure 2C). In contrast to TX01∆*trxH*, TX01∆*trxH*C and wild TX01 exhibited comparative capability of resistance against stresses caused by H_2_O_2_, acid, and Dp (Figure 2 and data not shown).

#### Effect on bacterial resistance to non-immune fish serum

**Figure 3:**
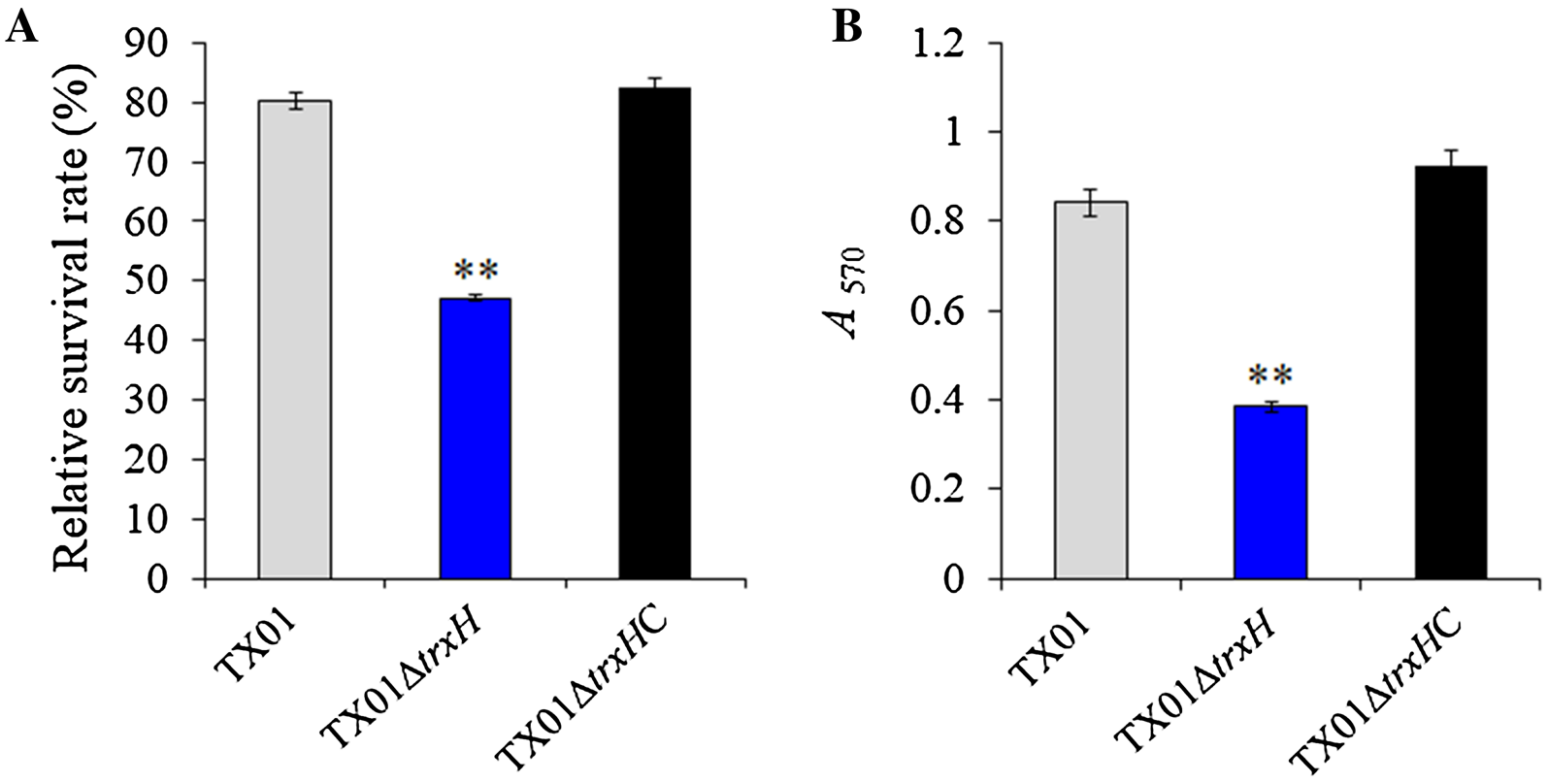
**Effects of** ***trxH***_***Ep***_ **mutation on resistance to serum and biofilm growth. A** Survival of *E. piscicida* in fish serum. TX01, TX01∆*trxH*, and TX01∆*trxH*C were incubated with non-immune flounder serum or PBS (control). After incubation, the survival of the bacteria was determined by plate counting. **B** The biofilm forming capacity of *E. piscicida*. TX01, TX01∆*trxH*, and TX01∆*trxH*C were incubated in polystyrene plate and biofilm formation was determined by measuring the the *A*_570_ of final eluates. Data are presented as mean ± SEM (*N* = 3). N, the number of times the experiment was performed. **P < 0.01.

Resistance against host serum killing is an important part of the virulence mechanism in many pathogens, and *E. piscicida* is known to resist the bactericidal effect of fish serum [14]. To examine whether *trxH*_*Ep*_ mutation affects the ability of serum tolerance, TX01, TX01∆*trxH*, and TX01∆*trxH*C were incubated with non-immune flounder serum for 1 h, and the survival of bacteria was determined by plate count. The result shows that TX01 and TX01∆*trxH*C exhibit apparent serum resistance, as 80.3% and 82.6% of the cells survived after incubation with flounder serum, respectively. However, TX01∆*trxH* displayed only 47.1% of surviving cells after serum treatment, which is significantly lower than for TX01 (Figure 3A).

#### Effect on biofilm formation

Since *trxH*_*Ep*_ mutations have no effect on planktonic growth of *E. piscicida* in normal conditions, we wanted to know whether bacterial biofilm formation was influenced by deletion of *trxH*_*Ep*_. The biofilm forming capacity of *E. piscicida* were analyzed and the result shows that biofilm growth of TX01∆*trxH* was significantly slower (2.2-fold less) than that of TX01. And the biofilm growth of TX01∆*trxH*C was similar to that of TX01 (Figure 3B).

#### Effect on cell invasion in vitro

**Figure 4:**
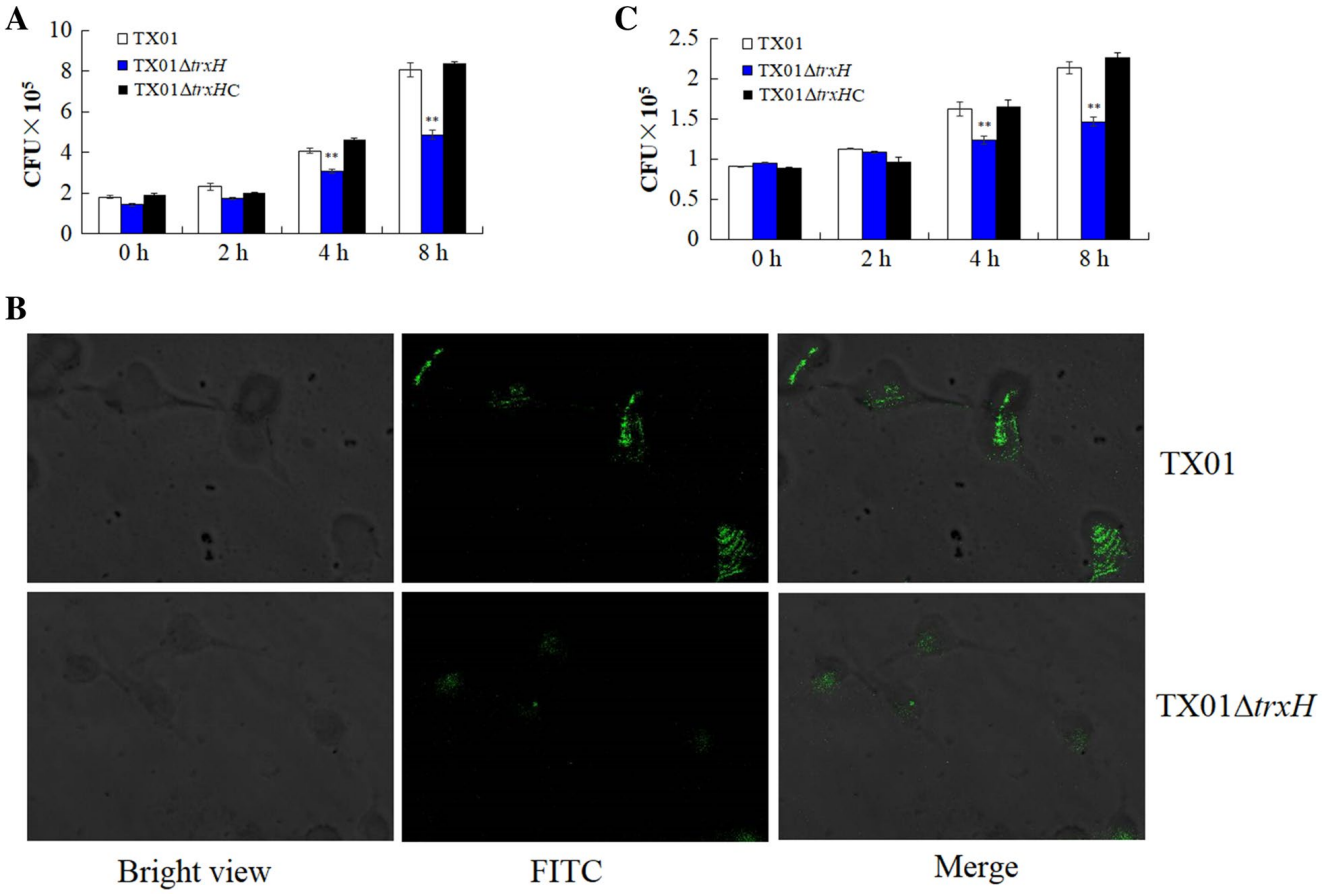
**Effect of** ***trxH***_***Ep***_ **mutation on cellular infection and replication. A** The invasion of *Edwardsiella piscicida* to flounder gill cells (FG cells). FG cells were infected with the same dose of TX01, TX01∆*trxH*, and TX01∆*trxH*C for various hours and washed with PBS. Then FG cells were lysed and the bacterial associated with and invaded into the host cells were determined. **B** FG cells were incubated with FITC-labeled *E. piscicida* TX01∆*trxH* or TX01, and the fluorescence of extracellular bacteria was quenched by adding trypan blue, then cells were observed with a confocal microscope. **C** Replication of *E. piscicida* in mouse macrophage cell RAW264.7. RAW264.7 cells were hatched with TX01, TX01∆*trxH*, and TX01∆*trxH*C for 2 h, then extracellular bacteria were killed. The cells were then incubated further for various hours, and the number of intracellular bacteria was determined by plate counting. Data are the means of three independent experiments and presented as mean ± SEM (*N* = 3). N, the number of times the experiment was performed. **P < 0.01.

To examine whether *trxH*_*Ep*_ mutation played any role in the infectivity of TX01, cultured FG cells were incubated with the same dose of TX01, TX01∆*trxH*, or TX01∆*trxH*C. The bacterial cells associated with infection and those invading the host cells were enumerated. The results show that the amount of TX01∆*trxH* recovered from FG cells was significantly lower than that of TX01 at 4 and 8 hpi, and the amount of TX01∆*trxH*C was comparative to that of TX01 (Figure 4A). Consistently, microscopic analysis shows that fluorescence intensity of cells infected by FITC-labeled TX01∆*trxH* was significantly weaker than that of cells infected by FITC-labeled TX01 (Figure 4B), indicating that the ability to infect the host cell was declined when *trxH*_*Ep*_ was inactivated.

It is known that *E. piscicida* is able to survive and replicate in the mouse macrophage cell line RAW264.7 [15]. To examine whether *trxH*_*Ep*_ mutation played any role in the intracellular survival of TX01, cultured RAW264.7 cells were incubated with the same dose of TX01 or TX01∆*trxH*, and extracellular bacteria were killed. The cells were then incubated further for various hours, and the number of intracellular bacteria was determined by plate count. The results show that the number of TX01∆*trxH* recovered from the intracellular RAW264.7 cells was significantly lower than those of TX01 at 4 and 8 hpi, and the amount of TX01∆*trxH*C was comparative to that of TX01 (Figure 4C).

#### Effect on bacterial dissemination in the fish tissues in vivo and general virulence

**Figure 5:**
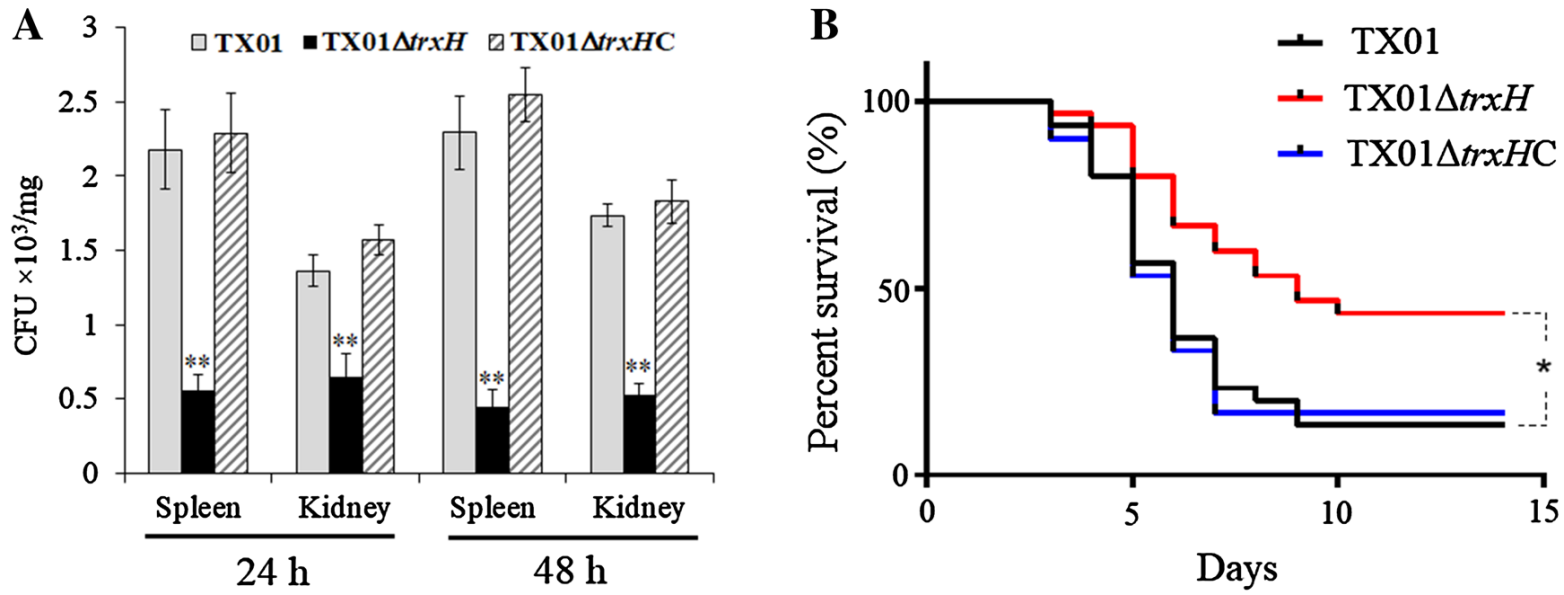
**In vivo infection of** ***Edwardsiella piscicida*** **in Japanese flounder. A** Bacterial dissemination in the fish tissues. Flounder were infected with the same dose of TX01, TX01∆*trxH*, or TX01∆*trxH*C, bacterial recoveries from spleen and kidney were determined by plate count at 24 and 48 hpi. **B** Host mortality induced by *E. piscicida*. Flounder were infected with equivalent doses of TX01, TX01∆*trxH*, and TX01∆*trxH*C, and accumulated mortality were monitored for a period of 20 days (only 15 days are shown since no more deaths occurred after 15 days). Significance between the survivals of wild type and mutant infected fish was determined with logrank test. Data are presented as means ± SEM (*N* = 3). N, the number of times the experiment was performed. *P < 0.05, **P < 0.01.

To examine the effect of *trxH*_*Ep*_ mutation on tissue infectivity, flounder were infected with the same dose of TX01, TX01∆*trxH*, or TX01∆*trxH*C, and bacterial recoveries from spleen and kidneys were determined at 24 and 48 hpi. The results show that bacterial recoveries from TX01∆*trxH*-infected fish were significantly lower than those from TX01-infected fish at 24 hpi and 48 hpi, and bacterial recoveries from TX01∆*trxH*C-infected fish were similar to those from TX01-infected fish (Figure 5A).

To examine the effect of *trxH*_*Ep*_ mutation on the overall bacterial virulence, Japanese flounder infected with TX01, TX01∆*trxH*, or TX01∆*trxH*C were used to monitor for mortality. The results show that at the end of the monitored period (20 days), the survival rate of TX01∆*trxH*-infected fish was 43.3%, which was significantly higher than that of TX01-infected fish (13.3%) (Figure 5B).

### Expression of *trxH*_*Ep*_, *trxA* and *trxC* in wild TX01 strain and mutants under different environmental conditions

#### Expression of trxH_Ep_ is modulated by stress

**Figure 6:**
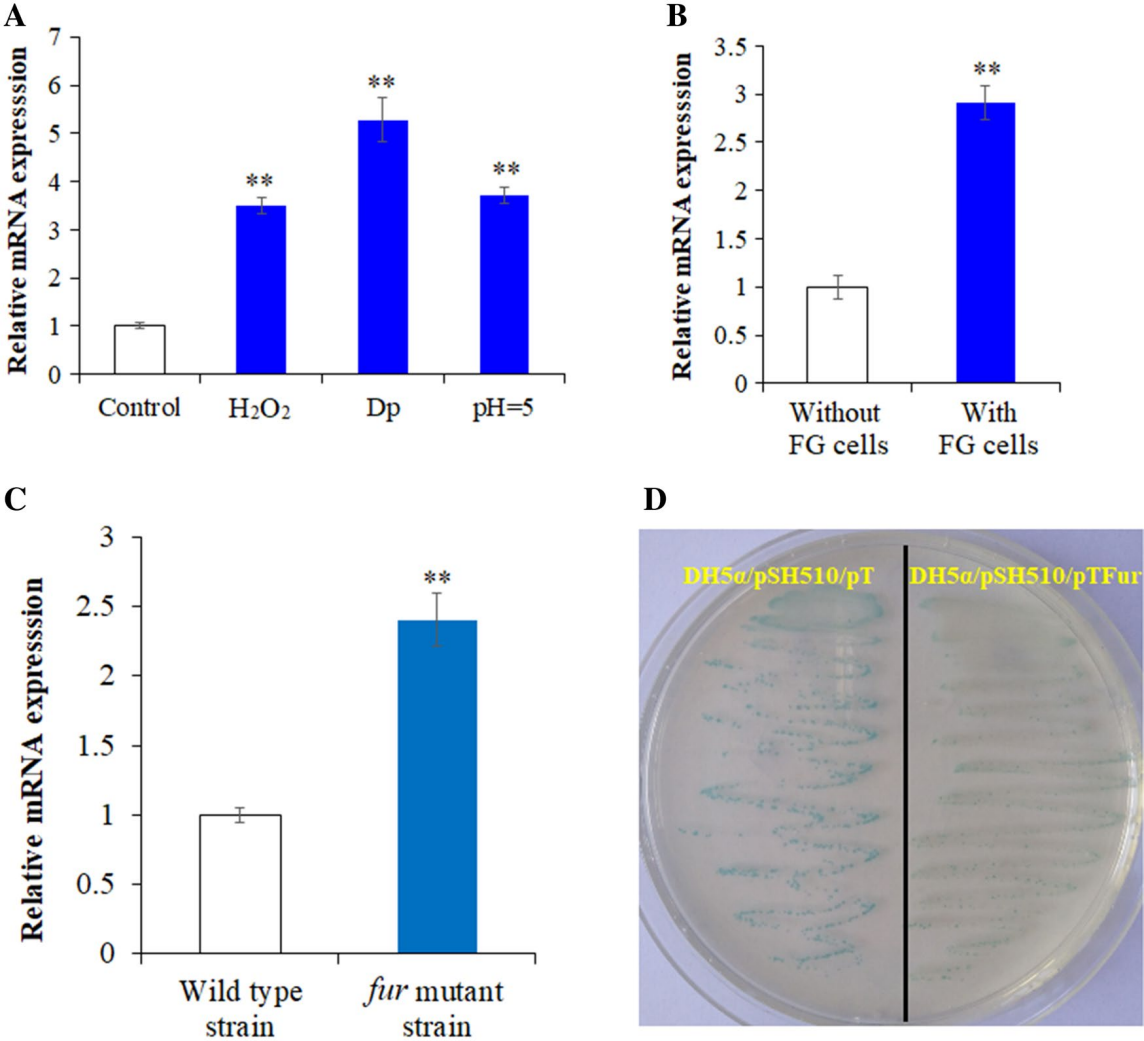
**Expression analysis of** ***trxH***_***Ep***_**. A** Expression of *trxH*_*Ep*_ under different conditions. RT-qPCR was performed with total RNA extracted from *Edwardsiella piscicida* TX01 cultured in normal LB medium (control, pH = 7), in hydrogen peroxide condition (normal LB medium with H_2_O_2_), in acid condition (pH = 5), in iron limitation condition (with the iron chelator 2,2′dipyridyl, Dp), Expression level of *trxH*_*Ep*_ in normal LB medium was set as 1. **B** RT-qPCR was performed with total RNA extracted from TX01 incubated with or without (control) FG cells. Expression level of *trxH*_*Ep*_ without FG cells was set as 1. **C** RT-qPCR was performed with total RNA extracted from wild type TX01 and *fur* mutant strain cultured in normal LB medium. Expression level of *trxH*_*Ep*_ in wild type TX01 strain was set as 1. **D** DH5α/pSH510/pTFur and DH5α/pSH510/pT were streaked and cultured on LB plate with X-gal, kanamycin, and ampicillin. Data are presented as mean ± SEM (*N* = 3). N, the number of times the experiment was performed. **P < 0.01.

Since thioredoxin plays a role in maintaining the intracellular redox homeostasis, and since it may participate in response to adversity, we determined the expression of *trxH*_*Ep*_ under normal conditions (i.e., cultured in LB medium), adverse conditions (i.e., cultured in LB medium with H_2_O_2_, Dp, or cultured in low pH LB medium), and infecting host cell condition by RT-qPCR. The results show that the expression of *trxH*_*Ep*_ was significantly enhanced when bacteria faced hydrogen peroxide pressure, acid pressure, iron deficiency, and an intracellular environment, compared to the expression of *trxH*_*Ep*_ in normal condition (Figures 6A and B).

#### Expression of trxH_Ep_ is regulated by Fur (Ferric uptake regulator)

In a previous study of *E. piscicida*, we obtained a *fur* mutant train, which exhibited much higher virulence than wild type *E. piscicida* strain TX01 (data not published). Proteomic analysis shows that the expression of TrxH was significantly up-regulated in the *fur* mutant strain, compared to the wild type strain (data not shown). So we detected the expression of *trxH*_*Ep*_ at the mRNA level. RT-qPCR shows that the expression of *trxH*_*Ep*_ in the *fur* mutant strain was 2.4-fold higher than that of *trxH*_*Ep*_ in the wild type strain (Figure 6C). Subsequently, we detected the regulatory effect of Fur on promoter activity of *trxH*_*Ep*_. The speculative promoter of *trxH*_*Ep*_, H510, was cloned to a promoter probe plasmid pSC11 and introduced into DH5α by transformation, resulting in DH5α/pSH510. When DH5α/pSH510 was cultured in LB agar plate with X-gal, the bacterial colonies were blue, which indicates that H510 has promoter activity. DH5α/pSH510 was then transformed with pTFur and pT (control). On the X-gal plate, the blue of DH5α/pSH510/pTFur was obviously weak, compared with DH5α/pSH510/pT (Figure 6D). β-Galactosidase assays show that galactosidase activity produced by DH5α/pSH510/pTFur was significantly lower (3.1 fold) than that produced by DH5α/pSH510/pT. These results indicate that Fur negatively regulated transcription of *trxH*_*Ep*_.

#### Expression of trxA and trxC in TX01∆trxH

**Figure 7:**
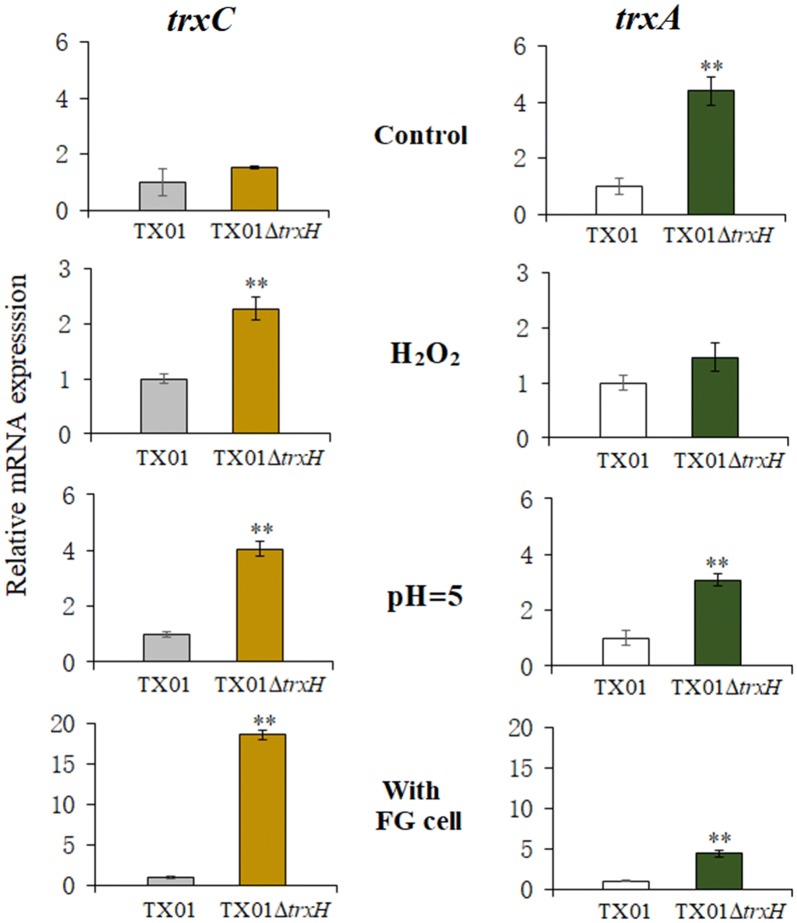
**Expression of** ***trxC*** **and** ***trxA*** **in wild type** ***Edwardsiella piscicida*** **TX01 strain and TX01∆*trxH*** **strain.** Total RNA was extracted from *E. piscicida* TX01 and TX01∆*trxH* cultured in normal LB medium (control, pH = 7), in hydrogen peroxide condition (with H_2_O_2_), in acid condition (pH = 5), or incubating with FG cells, respectively. Expressions of *trxC* and *trxA* were analyzed by RT-qPCR. Data are presented as mean ± SEM (*N* = 3). N, the number of times the experiment was performed. **P < 0.01.

There are two other thioredoxins, TrxA and TrxC, in *E. piscicida*, and their functions are unknown. To detect whether TrxA and TrxC undertake more functions when the TrxH function was deactivated, we examined the expression of *trxA* and *trxC* in TX01∆*trxH*. The results show that the deletion of *trxH* has no effect on the expression of *trxC* in normal conditions. However, when facing oxidative stress, acid pressure, or infecting host cell, the expression of *trxC* in TX01∆*trxH* was significantly up-regulated, compared to that in TX01. On the contrary, the deletion of *trxH* significantly induced 4.4-fold expression of *trxA* in normal conditions, but *trxA* expression exhibited no difference between TX01 and TX01∆*trxH* in the presence of hydrogen peroxide. Under an acidic environment and infecting host cell, the mRNA level of *trxA* in TX01∆*trxH* was 3.1- and 4.3-fold high, compared to the level of *trxA* in TX01 (Figure 7). These findings suggest that there is likely a complex relationship of functional complementation or expression regulation between TrxH and another two thioredoxins, TrxA and TrxC, of *E. piscicida*.

## Discussion

Thioredoxins play major roles in protection of cells against toxic oxygen species as well as maintaining the intracellular thio-disulfide balance in bacteria [42]. In *E. piscicida*, the function of thioredoxins is completely unknown. Moreover, we did not find any reports about the function of thioredoxin H in pathogen bacteria. In this study, a thioredoxin H homologue, TrxH, was identified and characterized from *E. piscicida*. TrxH_Ep_ contains an unusual motif (CPVC), not the typical Trx motif (CGPC), which is found in most Trx proteins. It is not known how this affects its activity or its roles.

It is reported that the expression of *trx* in many bacteria is induced by oxidative stress [20]. Similarly, in *E. piscicida*, the expression of *trxH*_*Ep*_ was significantly enhanced in the presence of H_2_O_2_. In other adverse conditions including acid stress and iron deficiency, *trxH*_*Ep*_ expression is upregulated. When bacteria infect host cells, the expression of *trxH*_*Ep*_ also increases. Under these conditions, the highest induction of *trxH*_*Ep*_ occured with iron deficiency, in which Fur exists predominately as a biologically inactive apoprotein [43]. Consistently, the expression of *trxH*_*Ep*_ in the *fur* mutant strain was higher than that in the wild type strain. As far as we know, this is the first report that Trx expression was regulated by Fur.

Besides TrxH, there are another two thioredoxin, TrxA and TrxC in *E. piscicida*. When *trxH*_*Ep*_ was inactivated, the expression of *trxC* did not change under normal conditions. However, under stress or infecting host cell conditions, *trxC* expression was significantly induced. As a chaperone, TrxC maybe undertook more roles in adverse conditions especially when *trxH* was inactivated in *E. piscicida*. On the contrary, the expression of *trxA* was directly or indirectly regulated by TrxH, which is sustained by the fact that Trx possesses the property of regulating gene expression [28]. However, in the presence of hydrogen peroxide *trxA* expression did not appear significantly different between wild TX01 and mutant TX01∆*trxH*. These findings show that there is likely to be a complex relationship between the three *trx* genes, and further research needs to be performed in the future.

Since the expression of *trxH*_*Ep*_ is induced by adverse conditions, it probably participates in stress resistance. Our results show that when *trxH*_*Ep*_ was inactive, the mutant TX01∆*trxH* exhibited a higher sensitivity to H_2_O_2_, which indicates that *trxH*_*Ep*_ plays an important role in tolerance to oxidative stress. Similar results were reported in other bacteria. In *H. pylori*, the ∆*trxC* mutant significantly decreased the ability of tolerance to oxygen exposure [24]. In *Listeria monocytogenes*, deletion of *trxA* markedly compromised tolerance of the pathogen to the thiol-specific oxidizing agent diamide [20]. Since Trx is involved in a series of cellular functions [26], we speculate that *trxH*_*Ep*_ participates in coping with other adversities, and our results confirm that *trxH*_*Ep*_ was involved in tolerance to acid stress and iron deficiency stress, which are similar to the adverse environment in the host.

It is generally known that Trx donates electrons to the disulfide bond system and helps isomerization of incorrectly paired disulfide bonds [20, 44]. Since many bacterial virulence factors require stable disulfide bonds for proper folding and function, Trx is closely related to infection of bacteria [45, 46]. It is reported that TrxA is essential for motility and contributes to host infection of *L. monocytogenes* [20]. In *H. pylori*, the ∆*trxC* mutant lacks mouse colonization ability, while the ability to colonize mouse stomachs is significantly reduced in the ∆trxA mutant [24]. In *Magnaporthe oryzae*, Trx1 and Trx2 also play important roles in bacterial pathogenesis [47]. In pathogenic fungus *Paracoccidioides lutzii*, Trx1 contributes to this organism’s virulence [19]. In this study, we also examined the effect of *trxH*_*Ep*_ on *E. piscicida* pathogenicity. It is known that biofilm formation and serum resistance are important parts of the virulence in multiple pathogens such as *E. piscicida* [17, 35] and *H. pylori* [48, 49]. In the latter, the inability to acquire iron from serum has been suggested to block *H. pylori* virulence by weakening its growth and survival in the serum [48]. Our results show that *trxH*_*Ep*_ mutation had no effect on growth of *E. piscicida*, but harmed bacterial biofilm growth. Similarly, the capability of serum resistance was significantly declined when *trxH*_*Ep*_ was inactivated. Consistently, upon infection with host cells, TX01∆*trxH* compared to TX01, shows an impaired ability to infect cells and to propagate in cells, indicating that *trxH* is required for bacterial invasion of host cells and host tissues. These observations suggest that *trxH*_*Ep*_ may play a role during bacterial invasion.

In conclusion, this study identified and characterized thioredoxin TrxH_Ep_ from fish pathogen *E. piscicida*. Our results indicate that TrxH_Ep_ possesses conservative thioredoxin motifs and that the *trxH*_*Ep*_ expression seems to be up-regulated under the pressure of several stressors, including iron deficiency. Regarding this fact, our results suggest the potential role of Fur for regulating *trxH*_*Ep*_ expression. TrxH_Ep_ not only played an important role in coping with adverse circumstances, but functioned as a factor that is essential to bacterial infection both at the cellular level and in a live fish model. This is the first study of TrxH in fish pathogen, and the results suggest that TrxH_Ep_ exerts pleiotropic effects on the pathogenesis of *E. piscicida*.
